# Relatedness coefficients in pedigrees with inbred founders

**DOI:** 10.1007/s00285-020-01505-x

**Published:** 2020-06-08

**Authors:** Magnus Dehli Vigeland

**Affiliations:** Department of Medical Genetics, University of Oslo, Oslo, Norway

**Keywords:** Relatedness, Kinship, Inbreeding, Identity coefficients, IBD triangle, Pedigree construction, 92D10, 92D25

## Abstract

We study an extension of the standard framework for pedigree analysis, in which we allow pedigree founders to be inbred. This solves a number of practical challenges in calculating coefficients of relatedness, including condensed identity coefficients. As a consequence we expand considerably the class of pedigrees for which such coefficients may be efficiently computed. An application of this is the modelling of background inbreeding as a continuous effect. We also use inbred founders to shed new light on *constructibility* of relatedness coefficients, i.e., the problem of finding a genealogy yielding a given set of coefficients. In particular, we show that any theoretically admissible coefficients for a pair of noninbred individuals can be produced by a finite pedigree with inbred founders. Coupled with our computational methods, implemented in the R package ribd, this allows for the first time computer analysis of general constructibility solutions, thus making them accessible for practical use.

## Introduction

A standard convention in pedigree analysis is that the pedigree founders are assumed to be noninbred. While this is natural in many settings, it is sometimes a severe limitation leading to unjustified approximations and biased results (Brustad and Egeland 2019; Kardos et al. 2018). In this paper we address this issue in the context of relatedness coefficients. We show that substantial benefits, both theoretical and practical, can be gained by relaxing the assumption of noninbred pedigree founders.

The purpose of relatedness coefficients is to quantify the amount of identical-by-descent (IBD) allele sharing between pedigree members. Alleles are said to be IBD if they have the same origin in some fixed reference population, typically the pedigree founders (Thompson 2013). It is important to distinguish the *pedigree-based* and *realised* coefficients; the former measure the *expected* IBD sharing, while the latter reflect the actual sharing in a given pair of individuals (Hill and Weir 2011). The realised coefficients can be estimated from genetic data, and may be preferable in certain situations (Speed and Balding 2015). However, family trees and pedigree coefficients continue to be a rich source of information. A recent example from human genetics is the massive undertaking by Kaplanis et al. (2018), where detailed pedigree-based coefficients were computed between millions of related individuals.

**Fig. 1:**
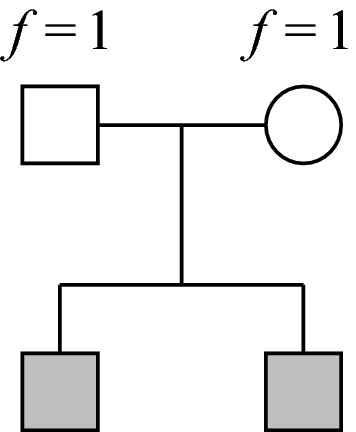
A pair of siblings whose parents are completely inbred. Current relatedness software struggle with such pedigrees because the inbred individuals require infinite mating chains for exact representation

A striking consequence of the conventional assumptions is that large classes of pedigrees are beyond reach of current software. For example, no software that we know of is capable of computing a complete set of IBD coefficients for the siblings shown in Fig. 1, whose parents are completely inbred. (This particular case is in fact trivial, since the two siblings are genetically like monozygotic twins.) In model organism experiments inbred strains are typically created by many generations of brother-sister mating or other breeding schemes. It is a mathematical fact, however, that the *inbreeding coefficientf* measuring the expected amount of IBD within an individual, will never reach 1 exactly; this is achievable only in the limit of an infinite pedigree. As a result it is impossible to represent completely inbred individuals in software following the standard conventions.

Another feature inadequately handled by existing methods is background inbreeding. This refers to the fact that all living creatures are related if their ancestry is traced far enough. Hence all individuals are in one sense inbred, including the designated founders of any real-life pedigree. It is well known that background inbreeding may seriously distort pedigree coefficients in wild pedigrees (see e.g. Kardos et al. 2018); nevertheless it is often ignored in practice. The problem is also present in human populations, where background inbreeding levels can rise to well over 5% (Pemberton and Rosenberg 2014; Leutenegger et al. 2011). To account for this, models incorporating background inbreeding have been proposed in various forms of pedigree analysis, e.g. in linkage analysis (Hössjer 2006) and genetic mapping of quantitative trait loci (Yi and Xu 2001).

The *kinship coefficient*, introduced by Wright (1922) almost 100 years ago, is the simplest measure of relatedness between two pedigree members. It is noteworthy that Wright’s famous path formula for this coefficient (see Eq. (1) below) explicitly incorporates founder inbreeding. Several previous authors, including Boichard (2002), and more recently Kirkpatrick et al. (2018), have published software allowing inbred founders in the computation of kinship coefficients.

In this work we extend the use of inbred founders to the full set of *condensed identity coefficients* (Jacquard 1966), which characterise in detail the expected genetic relationship between any two individuals. An efficient algorithm for computing these coefficients was first given by Karigl (1981), and generalised by other authors (Weeks and Lange 1988; Lange and Sinsheimer 1992). Karigl’s recursive approach remains popular due to its relative simplicity, but several alternative methods have been proposed over the years (Abney 2009; Cheng et al. 2009; García-Cortés 2015). We note especially the fast graphical algorithm implemented in the software IdCoefs (Abney 2009), also available through the R package identity, but none of these programs support inbred founders. To remedy this, we propose a modification of Karigl’s algorithm which accounts for arbitrary founder inbreeding.

The premise that pedigree founders may be inbred, but not related, is generally unrealistic under random mating. However, in specific cases our model assumptions are often supported by prior information about the founders. This applies in particular to pedigrees in medical and forensic genetics, where extensive data about the members are typically collected. For example, it may be known that the parents in a family are from different populations, and therefore unrelated. Importantly, such information may also be deduced from genetic data. Forensic pedigree analysis based on these ideas are explored in Brustad and Egeland (2019) and Vigeland and Egeland (2019).

When modelling background inbreeding in wild pedigrees, it is tempting to incorporate *founder relatedness* in addition to founder inbreeding. This idea has been pursued by Lacy (2012) in the case of kinship coefficients, and also in other forms of pedigree analysis (Sheehan and Egeland 2008). In the context of identity coefficients, the complexity of multi-person relatedness makes this generalisation out of scope for the present work, but perhaps not infeasible. We discuss this further in Section 6.1.

In this paper we apply pedigrees with inbred founders to take a fresh look at problems of *constructing* pedigree coefficients. This concerns the task of producing a genealogy yielding a prescribed set of coefficients, if at all possible. In addition to being theoretically attractive, such problems have considerable practical interest, for example in studies of ancient DNA (Prüfer et al. 2013). Constructibility of the full-blown identity coefficients remains elusive, but partial results have been found (Thompson 1980; Karigl 1984). In particular, Karigl (1984) gave a solution to the constructibility of pairwise identity coefficients in the case of noninbred individuals, employing a method for constructing arbitrary kinship coefficients. However, his constructions are difficult to work with, in general involving multiple infinite mating chains. Moreover, they are suboptimal in the sense that they *always* require infinite pedigrees, even in cases where finite solutions exist. Above all, his solutions are not suitable for computer implementation, thus effectively hindering researchers from analysing and experimenting with such pedigrees.

We provide alternative pedigree constructions rectifying the above issues. By allowing inbred founders, we show that any kinship coefficient, as well as any admissible set of IBD coefficients between noninbred individuals, can be produced by a finite pedigree. It should be emphasised that these theoretical results gain practical relevance from the computational methods presented in this paper. The algorithms are implemented in the R package ribd, enabling our constructions to be computer validated and used in practical examples. The ribd package is part of the ped suite of packages covering a wide range of pedigree analysis, with founder inbreeding as a core feature.

## Definitions and notation

We define a *pairwise relationship* to be a triple $(a,b,{\mathcal {P}})$, where ${\mathcal {P}}$ is a connected pedigree, and *a* and *b* are (not necessarily distinct) members of ${\mathcal {P}}$. Founders of ${\mathcal {P}}$, i.e., members whose parents are not included in ${\mathcal {P}}$, are assumed to be unrelated and noninbred unless explicitly stated otherwise. Homologous alleles of *a* and *b* are *identical by descent (IBD)* if they descend from the same allele carried by a common ancestor of *a* and *b* within ${\mathcal {P}}$. It should be emphasised that the concept of IBD, and consequently all coefficients to be defined below, depend on the context pedigree. We restrict our attention to diploid loci.

The simplest measure of relatedness between two pedigree members *a* and *b* is the *kinship coefficient*$\varphi _{ab}$, defined as the probability that a random allele from *a* is IBD with a random allele from *b* at the same locus. Moreover, if *a* and *b* have a child *c*, the *inbreeding coefficient*$f_c$ is the kinship coefficient of its parents, i.e., $f_c = \varphi _{ab}$. Equivalently, $f_c$ can be defined as the expected fraction of *c*’s autosomes that are *autozygous*, i.e., where the paternal and maternal alleles are IBD. The kinship/inbreeding coefficient was first studied by Wright (1922), who provided the following *path formula*:

1 $$\begin{aligned} \varphi _{ab} = \sum _s \sum _{u,v} \frac{1}{2^{|u| + |v| + 1}}(1 + f_s). \end{aligned}$$

The summation runs over all common ancestors *s* of *a* and *b*, and all pairs (*u*, *v*) of non-overlapping pedigree paths from *s* to *a* and *b* respectively, with path lengths |*u*| and |*v*|.

**Fig. 2:**
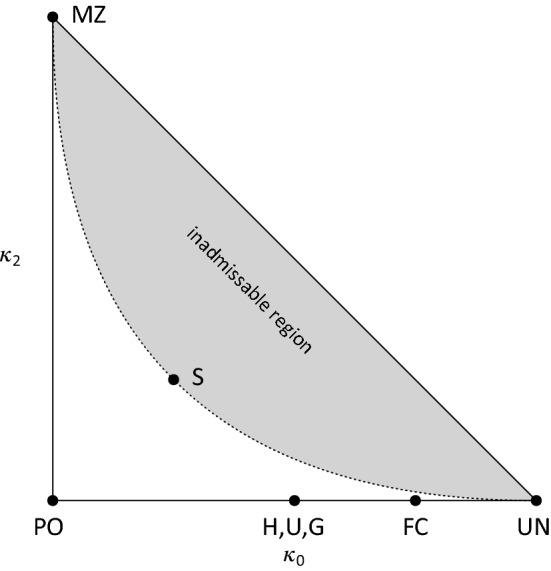
The IBD triangle. Each axis ranges from 0 to 1. Relationship abbreviations: FC = first cousins; G = grandparent-grandchild; H = half siblings; MZ = monozygotic twins; PO = parent-offspring; S = full siblings; U = avuncular (e.g. uncle-niece); UN = unrelated

For noninbred individuals *a* and *b* their *IBD coefficients*$\kappa =(\kappa _0, \kappa _1, \kappa _2)$ are defined as the probabilities of sharing respectively 0, 1 and 2 alleles IBD, at a random autosomal locus. Since $\kappa _0 + \kappa _1 + \kappa _2=1$, the triple $\kappa$ can be represented as a point $(\kappa _0, \kappa _2)$ in the *IBD triangle* shown in Fig. 2, defined by $\kappa _0, \kappa _2\ge 0$ and $\kappa _0+\kappa _2\le 1$. Thompson (1976) discovered that all relationships between noninbred individuals satisfy the inequality $\kappa _1^2 \ge 4\kappa _0\kappa _2$, thus defining an inadmissible region of the triangle, shown in grey in the figure. A point in the IBD triangle is called *admissible* if it is not in the inadmissible region. (Note that the boundary points are admissible.) Fig. 2 includes the location of some common outbred relationships.

**Fig. 3:**
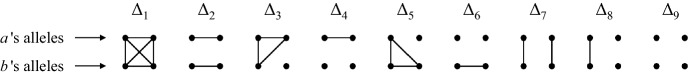
Jacquard’s condensed identity states and the corresponding coefficients. Each state represents a pattern of IBD between the alleles of individuals *a* and *b* at a single locus. IBD alleles are connected with a line segment

A complete characterisation of the expected IBD sharing at a single locus, of any two individuals, is given by the *condensed identity coefficients*$\varDelta _1, \dotsc , \varDelta _9$, attributable to Jacquard (1966). These are the expected relative frequencies of the *condensed identity states* shown in Fig. 3. The nine states represent the possible patterns of IBD between the four alleles carried by the two individuals, when the alleles within each individual are unordered. Importantly, when both individuals are noninbred, the first six states are impossible, and the remaining three correspond to the IBD coefficients in reverse order: $(\kappa _0, \kappa _1, \kappa _2) = (\varDelta _9, \varDelta _8, \varDelta _7)$.

## Identity coefficients in pedigrees with inbred founders

### A modification of Karigl’s algorithm

In this section we briefly review Karigl’s recursive algorithm for computing condensed identity coefficients (Karigl 1981), and we describe the modifications needed to accommodate inbred founders.

Define the *generalised kinship coefficient*$\varphi _{abc}$, for three (not necessarily distinct) pedigree members *a*, *b*, *c*, as the probability that if a random allele is sampled from each of them, at the same autosomal locus, all alleles are IBD. Similarly, we define $\varphi _{abcd}$ for 4 individuals. Finally let $\varphi _{ab,cd}$ be the probability that when homologous alleles are sampled randomly from *a*, *b*, *c*, *d*, the two from *a* and *b* are IBD and the two from *c* and *d* are IBD.

Karigl showed that the nine identity coefficients can be expressed as linear combinations of the generalised kinship coefficients defined above. The easiest way to see this is to start with the inverse relations. For example, for any individuals *a* and *b* we find by conditioning on the 9 identity states that

2 $$\begin{aligned} \varphi _{aab} = \varDelta _1 +\frac{1}{2}\varDelta _3+\frac{1}{4}\varDelta _5+\frac{1}{4}\varDelta _7+\frac{1}{8}\varDelta _8. \end{aligned}$$

From this and eight other similar identities a linear system of equations is obtained, which can be uniquely solved for $\varDelta , \dotsc , \varDelta _9$. We refer to Karigl (1981) for details.

For the computation of generalised kinship coefficients, Karigl (1981) gave the following recursion formulas, valid whenever *a* is a nonfounder with parents *p* and *m*, and *b*, *c*, *d* are (not necessarily distinct) nondescendants of *a*.

3 $$\begin{aligned} \begin{aligned} \varphi _{abc}&= \tfrac{1}{2}(\varphi _{pbc} + \varphi _{mbc})\\ \varphi _{aab}&= \tfrac{1}{2}(\varphi _{ab} + \varphi _{pmb})\\ \varphi _{aaa}&= \tfrac{1}{4}(1 + 3\varphi _{pm})\\ \varphi _{abcd}&= \tfrac{1}{2}(\varphi _{pbcd} + \varphi _{mbcd})\\ \varphi _{aabc}&= \tfrac{1}{2}(\varphi _{abc} + \varphi _{pmbc})\\ \varphi _{aaab}&= \tfrac{1}{4}(\varphi _{ab} + 3\varphi _{pmb})\\ \varphi _{aaaa}&= \tfrac{1}{8}(1 + 7\varphi _{pm})\\ \varphi _{ab,cd}&= \tfrac{1}{2}(\varphi _{pb,cd} + \varphi _{mb,cd})\\ \varphi _{aa,bc}&= \tfrac{1}{2}(\varphi _{bc} + \varphi _{pm,bc})\\ \varphi _{ab,ac}&= \tfrac{1}{4}(2\varphi _{abc} + \varphi _{pb,mc} + \varphi _{mb,pc})\\ \varphi _{aa,ab}&= \tfrac{1}{2}(\varphi _{ab} + \varphi _{pmb})\\ \varphi _{aa,aa}&= \tfrac{1}{4}(1 + 3\varphi _{pm})\\ \end{aligned} \end{aligned}$$

From the definitions it is clear that the generalised kinship coefficients are invariant under permutations of the indices, e.g. $\varphi _{abc} = \varphi _{bca}$ and $\varphi _{ab,cd} = \varphi _{ba,cd} = \varphi _{cd,ab}$ a.s.o. The boundary conditions are as follows: Whenever *a* and *b* are different founders (and *c* and *d* any members) the assumption of unrelatedness implies that

4 $$\begin{aligned} \varphi _{ab} = \varphi _{abc} = \varphi _{abcd} = \varphi _{ab,cd} = 0. \end{aligned}$$

Furthermore, under the assumption that all founders are outbred, elementary calculations show that

5 $$\begin{aligned} \begin{aligned} \varphi _{aaa}&= \tfrac{1}{4} \\ \varphi _{aaaa}&= \tfrac{1}{8} \\ \varphi _{aa,aa}&= \tfrac{1}{4} \\ \varphi _{aa,bb}&= \tfrac{1}{4}. \end{aligned} \end{aligned}$$

From the recursions (3) and boundary conditions (4) and (5) one can compute any generalised kinship coefficient involving up to four pedigree members, and thereby obtain the condensed identity coefficients as explained above.

Now we consider the situation when founders are allowed to be inbred. This has no impact on the general recursions (3); only the boundary values require modification. More precisely, the identities (5) must be replaced by the following formulas:

6 $$\begin{aligned} \begin{aligned} \varphi _{aaa}&= \tfrac{1}{4}(1 + 3f_a) \\ \varphi _{aaaa}&= \tfrac{1}{8}(1 + 7f_a) \\ \varphi _{aa,aa}&= \tfrac{1}{4}(1+3f_a) \\ \varphi _{aa,bb}&= \tfrac{1}{4}(1+f_a)(1+f_b) \end{aligned} \end{aligned}$$

Here $f_a$ and $f_b$ are the inbreeding coefficients of the founders *a* and *b* respectively. To verify the first of these formulas, suppose *i*, *j*, *k* are alleles sampled with replacement from *a*. We proceed by conditioning on the event that *a* is autozygous, i.e., that her alleles are IBD, which has probability $f_a$. Denoting this event by ${{\mathcal {A}}}$ we find

$$\begin{aligned} \begin{aligned} \varphi _{aaa}&= P(i,j,k \;\text {are IBD} \,|\,{\mathcal {A}})P({\mathcal {A}}) + P(i,j,k\; \text {are IBD} \,|\, {\mathcal {A}}^c)P({\mathcal {A}}^c) \\&= 1\cdot f_a + \tfrac{1}{4}(1-f_a) = \tfrac{1}{4}(1 + 3f_a) \end{aligned} \end{aligned}$$

as claimed. The remaining formulas in (6) are proved similarly.

### Implementation

We have implemented the modified algorithm presented in the previous section in the R package ribd, which is freely available (https://CRAN.R-project.org/package=ribd). In addition to the nine identity coefficients, the package offers separate functions for generalised kinship coefficients, IBD coefficients (of noninbred individuals), and standard kinship coefficients. Founder inbreeding is allowed in all cases. Furthermore, ribd contains algorithms for computing X-chromosomal kinship and identity coefficients, as well as various two-locus coefficients.

## Effects of background inbreeding on IBD coefficients

**Fig. 4:**
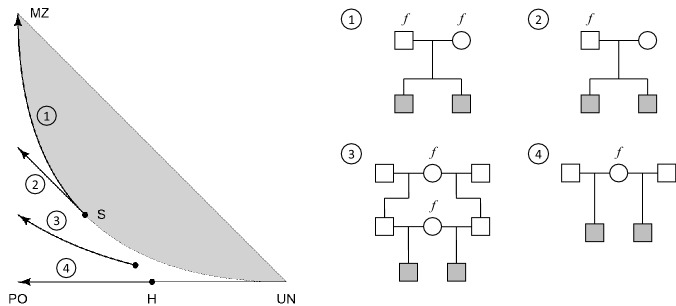
The effect of founder inbreeding in full sib and a selection of full-and half-sib relationships. Each arrow traces the IBD coefficients as the level of founder inbreeding increases from 0 to 1

The presence of inbreeding in pedigree founders can have a large effect on the genetic relationships within the pedigree. Fig. 4 illustrates this for a selection of sibling relationships. Note that in each pedigree the siblings are noninbred, and remain so even if the founders are inbred; hence the IBD coefficients are well defined in all cases and fully characterise the relationships. The arrows trace the IBD coefficients of the sibs as the background inbreeding level *f* increases from 0 to 1. For example, the first arrow shows that full siblings become indistinguishable from monozygotic twins when both parents are completely inbred. Similarly, the half siblings in pedigree four will appear as (outbred) parent-offspring if their shared parent is inbred.

An interesting feature of founder inbreeding is exemplified by pedigrees 1 and 2 in Fig. 4. In the first of these, both parents are assigned the same background inbreeding level, while in the second, only the father is inbred. The impact on the IBD coefficients is quite different in the two cases, as can be seen by the corresponding arrows. If both of the parental inbreeding coefficients are allowed to vary freely, the resulting IBD coefficients of the siblings cover the entire region between arrows 1 and 2.

## Constructibility theorems

In this section we improve on two results of Karigl regarding the constructibility of kinship and IBD coefficients. The main idea is to use inbred founders to mask most of the complexity. It turns out that this can always be carried out in such a way that a finite pedigree suffices. As a consequence, all of our constructions may be analysed and verified with the ribd package.

### Constructibility of kinship coefficients

The following theorem is due to Karigl (1984):

#### Theorem A

(Karigl) Any number $\varphi \in [0,1]$ is constructible as a kinship coefficient in the limit of an infinite pedigree.

What Karigl actually proved was that for any $\varphi$ there exists a *finite* pedigree with individuals *a* and *b* such that $\varphi _{ab}$ is *arbitrarily close* (but never equal) to $\varphi$. Only by extending his mating scheme *ad infinitum*, can $\varphi$ be generated exactly. In fact, his construction contains two infinite parts: One needed to generate a completely inbred individual (e.g. by an infinite chain of sib-mating), and another involving repeated backcrosses. The latter part can be made finite if $\varphi$ is a dyadic fraction, but never the first; hence the construction always produces an infinite pedigree.

We now introduce a class of simple relationships, which we will use repeatedly in what follows. Essentially, these are half-cousin relationships, but where we allow the shared ancestor to be inbred. As we will see, this enables the construction of any kinship coefficient, but with the crucial advantage of hiding all the infinite parts in a single founder inbreeding coefficient.

#### Definition 1

Members *a* and *b* of a pedigree ${\mathcal {P}}$ are called *half cousins with founder inbreedingf* if they are connected through a single non-collapsing path in ${\mathcal {P}}$, and the top-most path member has inbreeding coefficient *f*. The path length is called the *separation* of *a* of *b*.

**Fig. 5:**
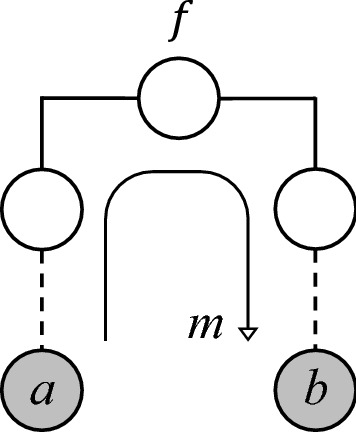
Half cousins with separation *m* and founder inbreeding *f*

The actual genealogy of the inbred path member, i.e., how the inbreeding coefficient *f* was produced, is irrelevant for our purposes of computing relatedness coefficients. Hence we regard this individual as a founder with an assigned inbreeding coefficient, as illustrated in Fig. 5. (Some limitations of this approach are discussed in Sect. 6.2.) Note that in Fig. 5, and all other pedigree drawings in this paper, the founders not shown are assumed to be outbred and unrelated.

We denote by ${{\mathcal {H}}}{{\mathcal {C}}}_m^f$ the set of half cousin relationships with separation *m* and founder inbreeding *f*, and no further relationships or inbreeding involving the path members. It is convenient to include in this notation the cases $m=0$ (corresponding to $a=b$, or identical twins) and $m=\infty$ (infinitely distant half cousins). Observe that for $m>1$ the set ${{\mathcal {H}}}{{\mathcal {C}}}_m^f$ contains pedigrees of different structures. For example, ${{\mathcal {H}}}{{\mathcal {C}}}_2^0$ contains half siblings, but also grandparent/grandchild. In contrast, the set ${{\mathcal {H}}}{{\mathcal {C}}}_1^f$ has essentially only one element (ignoring gender swaps), namely a parent/child relationship where the parent has inbreeding coefficient *f*.

At first glance half cousins may seem like a small class of relationships. But as the next theorem shows, they in fact cover the entire spectrum of kinship coefficients:

#### Theorem 2

Any number $\varphi \in [0,1]$ is constructible as the kinship coefficient of a half cousin relationship with inbred founder.

**Fig. 6:**
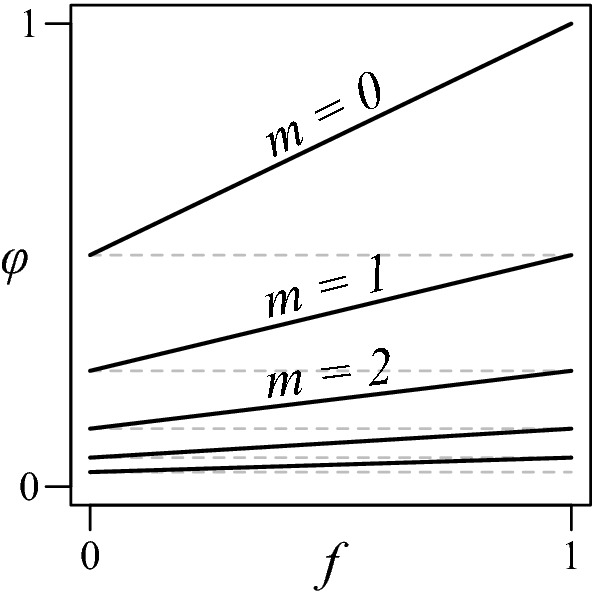
Dissection of the kinship formula for half cousin relationships, $\varphi = 2^{-m-1}(1+f)$

#### Proof

Observe that when *m* is finite, the kinship coefficient of any half cousin relationship $(a,b,{\mathcal {P}}) \in {{\mathcal {H}}}{{\mathcal {C}}}_m^f$ is given by Wright’s formula (1) to be

7 $$\begin{aligned} \varphi _{ab} = \frac{1}{2^{m+1}}(1+f). \end{aligned}$$

For a fixed *m* the map $f\mapsto \frac{1}{2^{m+1}}(1+f)$ maps the unit interval [0, 1] bijectively to $[\frac{1}{2^{m+1}},\frac{1}{2^m}]$. When *m* runs through $0,1,\dotsc$ these intervals cover the entire (0, 1], as illustrated in Fig. 6. An inverse map is given by

8 $$\begin{aligned} \begin{aligned} m&= \lceil \log _2\frac{1}{\varphi } \rceil - 1 \\ f&= \varphi 2^{m+1} - 1, \end{aligned} \end{aligned}$$

when $\varphi \in (0,1)$, and $(m,f) = (0,1)$ when $\varphi = 1$. In fact *m* and *f* are uniquely determined by $\varphi$, except when $\varphi = 2^{-k}$ for some $k\in {\mathbb {N}}$; in this case both $(m,f) = (k,0)$ and $(m,f) = (k-1,1)$ will do.

Finally, the endpoint $\varphi =0$ corresponds to $m=\infty$, i.e., infinitely distant half cousins. In this case the value of *f* is irrelevant. $\square$

We emphasise that Theorem 2 does not imply Theorem A. Such a leap would require the existence of an individual with arbitrary inbreeding coefficient, leading to a circular argument. However, we now give an independent proof of existence. In fact, the following is an improved version of Theorem A, in the sense that our construction provides a finite pedigree whenever this is theoretically possible (when $\varphi$ is a dyadic fraction), and at most one infinite chain in the general case.

#### Theorem 3

Any number $\varphi \in [0,1]$ is constructible as a kinship coefficient in a (possibly infinite) chain of half cousin relationships with outbred founders.

#### Proof

The endpoint $\varphi =0$ is solved by ${{\mathcal {H}}}{{\mathcal {C}}}_\infty ^0$, so we can assume $\varphi >0$. By taking a binary representation $\varphi =0.r_1r_2...$, and letting $s_1, s_2, \dotsc$ be the indices of the 1’s, we can write $\varphi$ as a sum of different negative powers of 2:

$$\begin{aligned} \varphi = \sum 2^{-s_i}, \qquad 1 \le s_1< s_2 < \cdots \end{aligned}$$

The sum can be made finite if and only if $\varphi$ is a dyadic fraction. Let $t_1, t_2, \dotsc$ be the increments of the $s_i$’s, i.e., $t_1=s_1$, $t_2=s_2-s_1$ a.s.o., so that $t_1+\cdots +t_k=s_k$ for all $k\in {\mathbb {N}}$.

For each $i=1,2,\dotsc$, choose $(a_i, b_i, {\mathcal {P}}_i) \in {{\mathcal {H}}}{{\mathcal {C}}}_{t_i - 1}^0$, and consider the pedigree ${\mathcal {P}}^*$ formed by chaining ${\mathcal {P}}_1, {\mathcal {P}}_2, \dotsc$ such that the founder of ${\mathcal {P}}_i$ becomes a child of $a_{i+1}$ and $b_{i+1}$. By repeated use of the formula (7) we find that within ${\mathcal {P}}^*$ the kinship coefficient between the bottom individuals is

$$\begin{aligned} \varphi _{a_1b_1}&= 2^{-t_1}(1 + 2^{-t_2}(1 + 2^{-t_3}(1 + \cdots ))) \\&= 2^{-t_1} + 2^{-(t_1 + t_2)} + 2^{-(t_1 + t_2 + t_3)} +\cdots \\&= 2^{-s_1} + 2^{-s_2} + 2^{-s_3} +\cdots \\&= \varphi . \end{aligned}$$

$$\square $$

#### Example 4

**Fig. 7:**
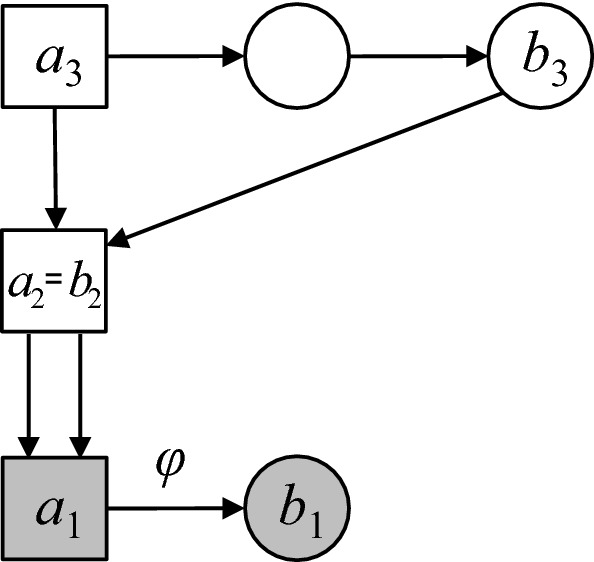
A relationship with kinship coefficient $\varphi = 1/2^2 + 1/2^3 + 1/2^6$. Each arrow represents a parent-child relationship

Fig. 7 shows an example of the construction for the kinship coefficient $\varphi = 0.390625 = 1/2^2 + 1/2^3 + 1/2^6$. The exponent sequence $\{2, 3, 6\}$ has increments $t = \{2,1,3\}$, hence the layers are elements of ${{\mathcal {H}}}{{\mathcal {C}}}_{1}^0$, ${{\mathcal {H}}}{{\mathcal {C}}}_{0}^0$, ${{\mathcal {H}}}{{\mathcal {C}}}_{2}^0$ respectively, starting from the bottom. Note that the construction requires selfing whenever $t_i=1$.

We end this section with an observation about breeding schemes with stationary inbreeding levels.

#### Proposition 5

For each $m\in {\mathbb {N}}_0$ there is a unique $f^*\in (0,1]$ such that, if *a* and *b* are half cousins with separation *m* and founder inbreeding $f^*$, then $\varphi _{ab} = f^*$.

#### Proof

The point is that $f^*$ must be a fixed point of the map $f\mapsto \frac{1}{2^{m+1}}(1+f)$. It is straightforward to show that such $f^*$ exists and is unique for each *m*, with value

$$\begin{aligned} f^* = \frac{1}{2^{m+1}-1}. \end{aligned}$$

$$\square $$

**Fig. 8:**
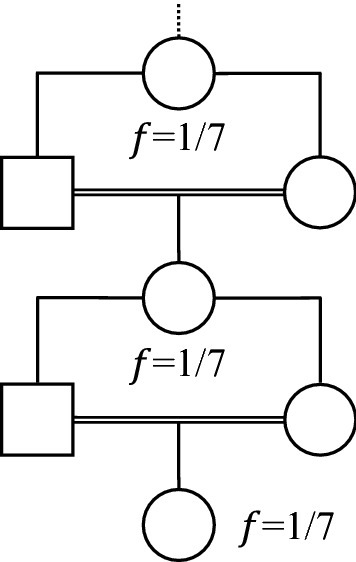
A mating scheme with stationary inbreeding level

An example is shown in Fig. 8, where sequential half-sib matings maintain a stationary inbreeding coefficient of $f = 1/7$.

### Constructibility of IBD coefficients

Karigl’s constructibility theorem for IBD coefficients can be stated, in our terminology, as follows (Karigl 1984):

#### Theorem B

(Karigl) Any admissible point in the IBD triangle is constructible as IBD coefficients in the limit of an infinite pedigree.

Karigl’s proof of this theorem relies on a combination of several limit processes. The resulting pedigree in general contains 4 infinite parts, making it unsuitable for computer implementation. In contrast, by using half cousins with inbred founders, we are able to give a different construction which is always finite, and can be easily analysed in appropriate software.

**Fig. 9:**
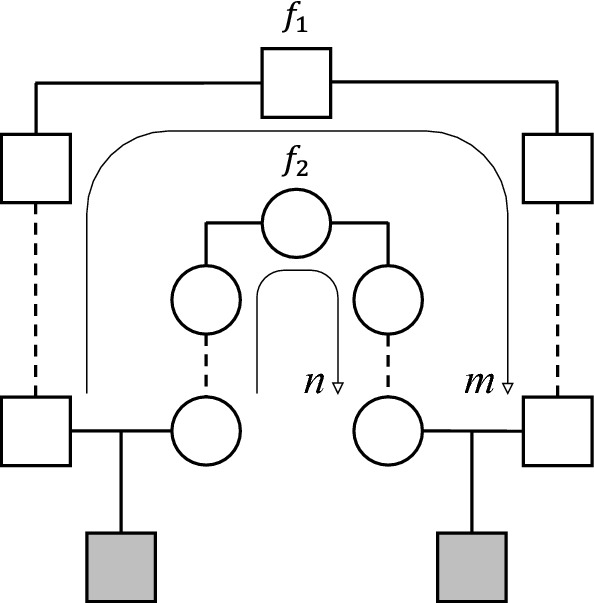
Double half cousins with separations *m* and *n*, and founder inbreeding $f_1$ and $f_2$

#### Definition 6

Let *m*, *n* be nonnegative integers, and $f_1, f_2$ numbers in the interval [0, 1]. Then let $\mathcal {DHC}_{m, n}^{f_1, f_2}$ denote the class of relationships between two individuals such that the fathers are half cousins ${{\mathcal {H}}}{{\mathcal {C}}}_m^{f_1}$, the mothers are half cousins ${{\mathcal {H}}}{{\mathcal {C}}}_n^{f_2}$, and there are no further relationships or inbreeding.

The definition is illustrated in Fig. 9. Note that the definition implies that the fathers are not related to the mothers; hence neither of the two bottom individuals are inbred.

#### Theorem 7

Any admissible point in the IBD triangle is constructible as a double half cousin relationship with inbred founders.

#### Proof

Consider two individuals *a* and *b* whose fathers have kinship coefficient $\varphi _{1}$ and whose mothers have kinship coefficient $\varphi _{2}$. Without loss of generality we can assume $\varphi _{1}\le \varphi _{2}$. Suppose further that the fathers are unrelated to the mothers. Then it follows that *a* and *b* are noninbred, and that their IBD coefficients are given by

9 $$\begin{aligned} \begin{aligned} \kappa _0&= (1-\varphi _{1})(1-\varphi _{2}) \\ \kappa _2&= \varphi _{1}\varphi _{2}. \end{aligned} \end{aligned}$$

As observed by Thompson (1976) these equations can always be solved for $\varphi _{1}$ and $\varphi _{2}$ when $(\kappa _0, \kappa _2)$ is in the admissible region. In explicit terms, a little algebra shows that $\varphi _{1}$ and $\varphi _{2}$ satisfy the quadratic equation $\varphi ^2 - (1-\kappa _0+\kappa _2)\varphi + 4\kappa _2=0$, which has solutions

10 $$\begin{aligned} \begin{aligned} \varphi _{1}&= \frac{1}{2}(U - \sqrt{D}) \\ \varphi _{2}&=\frac{1}{2}(U + \sqrt{D}), \end{aligned} \end{aligned}$$

where $U = 1 + \kappa _2 - \kappa _0$ and $D=U^2-4\kappa _2$. Note that the discriminant $D=U^2-4\kappa _2 = \kappa _1^2-4\kappa _0\kappa _2$ is nonnegative if and only if $\kappa$ is admissible. Furthermore, since $D \le U^2$ we have $\varphi _{1}\ge 0$, and similarly $D\le \kappa _1^2$ gives $\varphi _{2} \le \frac{1}{2}(U + \kappa _1)= 1 - \kappa _0 \le 1$. Hence for any admissible $\kappa$ the solutions (10) are well-defined and satisfy $0\le \varphi _{1} \le \varphi _{2} \le 1$.

The point is now that by Theorem 2, $\varphi _{1}$ and $\varphi _{2}$ can be constructed as kinship coefficients by means of half cousin relationships ${{\mathcal {H}}}{{\mathcal {C}}}_m^{f_1}$ and ${{\mathcal {H}}}{{\mathcal {C}}}_n^{f_2}$ respectively, for suitable values of $m,n,f_1,f_2$. But this means precisely that *a* and *b* are double half cousins in $\mathcal {DHC}_{m, n}^{f_1, f_2}$.

For explicit values of $m,n,f_1,f_2$ we insert (10) into the formulas (8) in the proof of Theorem 2:

11 $$\begin{aligned} \begin{aligned} m&= \left\lceil \log _2 \frac{1}{U - \sqrt{D}} \right\rceil ,&n&= \left\lceil \log _2 \frac{1}{U + \sqrt{D}} \right\rceil , \\ f_1&= 2^m(U - \sqrt{D}) - 1,&f_2&= 2^n(U + \sqrt{D}) - 1. \end{aligned} \end{aligned}$$

The above formulas are valid and well-defined when $0< \varphi _1, \varphi _2 < 1$. The edge cases are dealt with as follows: If $\varphi _1 = 1$ we take $m = 0$ and $f_1 = 1$; similarly $\varphi _2 = 1$ gives $n = 0$ and $f_2 = 1$. When $\varphi _{1}=0$ then Theorem 2 yields $m=\infty$, while $f_1$ can take any value in [0, 1]. Finally, if $\varphi _{2}$ is also 0, a solution is given by $m=n=\infty$ (and $f_1, f_2$ any values in [0, 1]). This concludes the proof. $\square$

Note that if the fathers in a double half cousin relationship have infinite separation ($m=\infty$), they are in fact unrelated. The relationship then reduces to (maternal) half cousins, so we can write $\mathcal {DHC}_{\infty , n}^{f_1, f_2} = {{\mathcal {H}}}{{\mathcal {C}}}_n^{f_2}$. This simple observation enables us to re-formulate the previous theorem into the following important result:

#### Theorem 8

If inbred founders are allowed, any admissible point in the IBD triangle is constructible in a finite pedigree.

#### Proof

Suppose $\kappa$ is an admissible point. If $\kappa _2 > 0$, then the proof of Theorem 7 yields finite values of *m* and *n*. If $\kappa _2 = 0$, we obtain $m=\infty$, but as explained above this can be viewed as a half cousin relationship ${{\mathcal {H}}}{{\mathcal {C}}}_n^{f_2}$. Finally, the vertex $\kappa = (1,0,0)$ is trivially constructible: any pedigree containing two noninbred founders will suffice. $\square$

We now go on to describe a subdivision of the admissible region which results from, and illuminates, our construction. For a fixed choice of $m,n \in {\mathbb {N}}_0 \cup \{\infty \}$, let $A_{m,n}$ be the subset of points in the IBD triangle constructible by double half cousins with separations *m* and *n*, i.e., relationships in $\mathcal {DHC}_{m, n}^{f_1, f_2}$ for varying $f_1, f_2$. Clearly $A_{m,n} = A_{n,m}$, so to avoid redundancy we assume from now on that $m\ge n$. The next theorem shows that each $A_{m,n}$ is a closed subset, and implies that they in collection form a subdivision of the admissible region. To prepare the statement, let $v_{i,j}$ denote the point with coordinates

$$\begin{aligned} v_{i,j} = \bigl ((1-\tfrac{1}{2^i})(1-\tfrac{1}{2^j}), \; \tfrac{1}{2^i}\tfrac{1}{2^j} \bigr ), \end{aligned}$$

for any $i,j \in {\mathbb {N}}_0$. To include infinite indices we set $v_{\infty , j} = v_{j, \infty } = (1-\frac{1}{2^j}, 0)$ and $v_{\infty , \infty } = (1, 0)$. We define $V_{i,j}$ to be the convex hull of $v_{i,j}$, $v_{i+1,j}$, $v_{i,j+1}$, $v_{i+1,j+1}$.

#### Theorem 9

The set $A_{m,n}$ is the intersection of the admissible region with $V_{m,n}$.

#### Proof

Suppose first that $\kappa = (\kappa _0, \kappa _2) \in A_{m,n}$, i.e., that $\kappa$ is the IBD coefficients of some relationship $(a,b,{\mathcal {P}})\in \mathcal {DHC}_{m,n}^{f_1,f_2}$. Combining Eqs. (7) and (9) we obtain

12 $$\begin{aligned} \begin{aligned} \kappa _0&= \bigl (1-\frac{1+f_1}{2^{m+1}}\bigr ) \cdot \bigl (1-\frac{1+f_2}{2^{n+1}}\bigr ) \\ \kappa _2&= \frac{1+f_1}{2^{m+1}}\cdot \frac{1+f_2}{2^{n+1}}. \end{aligned} \end{aligned}$$

By straightforward manipulation of these expressions it can be verified that

13 $$\begin{aligned} \kappa = f_1f_2v_{m,n} + f_1{\overline{f}}_2v_{m,n+1} + {\overline{f}}_1f_2v_{m+1,n} +{\overline{f}}_1{\overline{f}}_2v_{m+1,n+1}, \end{aligned}$$

where ${\overline{f}}_1=1-f_1$ and ${\overline{f}}_2=1-f_2$. This shows that $A_{m,n} \subseteq V_{m,n}$.

**Fig. 10:**
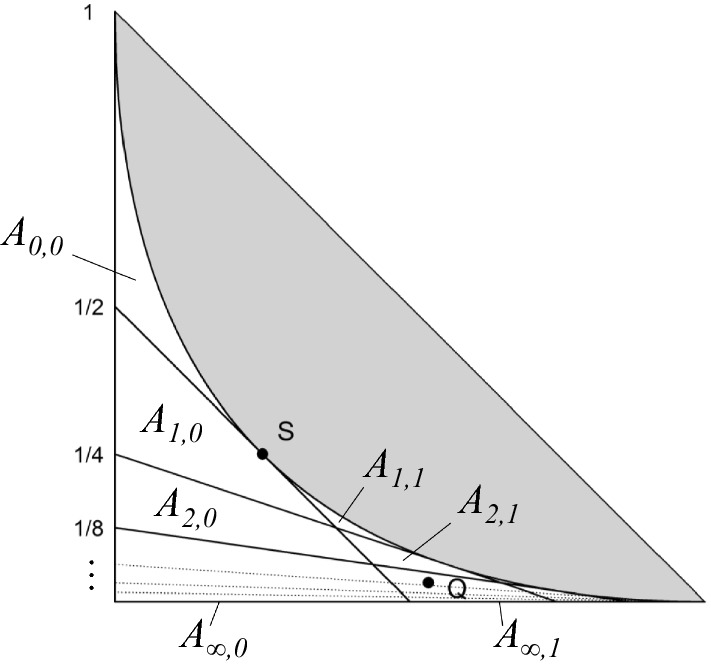
The subdivision of the admissible region described in Theorem 9. The points *S* and *Q* are examined in Examples 10 and 11 respectively

Conversely, suppose $\kappa$ is an admissible point in the interior of $V_{m,n}$. By Theorem 7$\kappa$ is constructible by a relationship of type $\mathcal {DHC}_{m',n'}^{f'_1,f'_2}$ for some $m',n',f'_1,f'_2$. The previous argument then shows that $\kappa \in V_{m',n'}$. But it is easy to check that $V_{m,n}$ and $V_{m',n'}$ have disjoint interiors if $(m,n) \ne (m',n')$. Thus, since $\kappa$ was assumed to be in the interior of $V_{m,n}$, we must have $m=m'$ and $n=n'$; in other words $\kappa \in A_{m,n}$.

Finally, suppose $\kappa$ is on the boundary of $V_{m,n}$, say, on the edge connecting $v_{m,n}$ and $v_{m, n+1}$. Then $\kappa = \lambda v_{m,n} + (1-\lambda ) v_{m,n+1}$ for some $\lambda \in [0,1]$. Setting $f_1 = 1$ and $f_2 = \lambda$ in (13) it is clear that $\kappa$ is constructible by $\mathcal {DHC}_{m,n}^{1, \lambda }$. In particular, this means that $\kappa \in A_{m,n}$. The other edges are proved similarly. This concludes the proof. $\square$

Figure 10 shows the subdivision induced by varying *m* and *n*. Note that when *m* and *n* are finite, $A_{m,n}$ is a quadrangle if $m>n+1$, a triangle if $m=n+1$, and a parabolic subspace if $m=n$. In the limit we find that $A_{\infty , n}$ is a line segment on the bottom edge of the triangle, and $A_{\infty , \infty }$ is the vertex (1, 0) corresponding to unrelatedness.

Within the interior of each $A_{m,n}$ the values of $m,n,f_1,f_2$ are uniquely determined by $\kappa$. When $\kappa$ lies on the border between two or more regions, however, multiple realisations are possible.

**Fig. 11:**
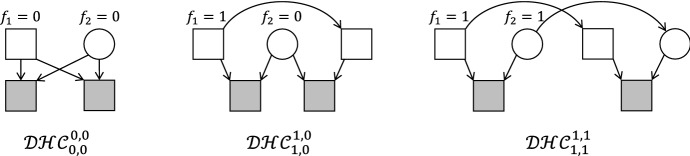
Three constructions of the IBD coefficients $\kappa = (\frac{1}{4}, \frac{1}{2}, \frac{1}{4})$. Left: Full siblings. Middle: Maternal half siblings whose fathers are father-and-son, and the elder father is completely inbred. Right: A double half avuncular relationship, where both common ancestors are completely inbred

#### Example 10

The point $S = (\frac{1}{4}, \frac{1}{4})$ in the IBD triangle is normally associated with the relationship between outbred, full siblings. As seen in Fig. 10, however, *S* in fact belongs to three regions: $A_{0,0}$, $A_{1,0}$ and $A_{1,1}$. Each of these give rise to fundamentally different genealogies producing the IBD coefficients $(\kappa _0, \kappa _2) = S$. These are illustrated in Fig. 11.

For our final example we turn to a popular case in the literature of pedigree analysis, namely the relationship of quadruple half first cousins. It is well known that the IBD coefficients of this relationship are $\kappa =(\frac{17}{32},\frac{14}{32},\frac{1}{32})$, corresponding to the point *Q* in Fig. 10 (see e.g. Thompson 2000). To the best of our knowledge the following is the first known example of a *different* relationship with exactly these IBD coefficients.

**Fig. 12:**
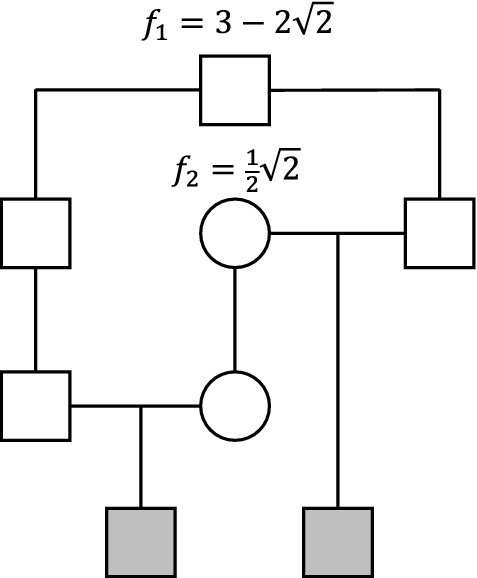
A relationship with the same IBD coefficients as quadruple half first cousins

#### Example 11

By Theorem 7, the coefficients $\kappa =(\frac{17}{32},\frac{14}{32},\frac{1}{32})$ are constructible as a double half cousin relationship. To find the separations and founder inbreeding coefficients first observe that $U = \frac{1}{2}$ and $D = \frac{1}{8}$, where *U* and *D* are defined in the proof of Theorem 7, and then use the formulas (11) to compute $m=3$, $n=1$, $f_1 = 3 - 2\sqrt{2}$ and $f_2 = \frac{1}{2}\sqrt{2}$. The values for *m* and *n* imply that *Q* lies in the region $A_{3,1}$, which is in agreement with Fig. 10. An explicit construction is shown in Fig. 12, where we have chosen the fathers to be half first cousins once removed ($m=3$), while the mothers are mother-and-daughter ($n=1$).

We can verify Example 11 computationally in R with the ribd package as follows:
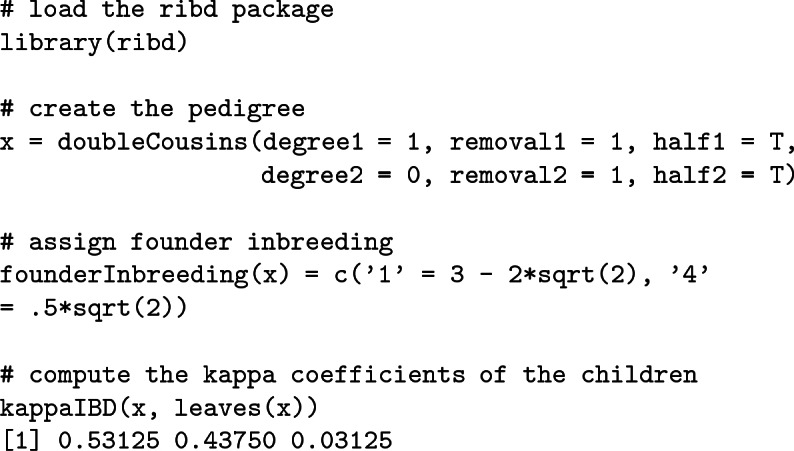


## Discussion

The most immediate consequence of this work and its implementation in ribd, is a substantial expansion of the class of pedigrees for which identity coefficients can be exactly computed. This pertains to any pedigree containing a completely inbred individual, or in fact, any member with a non-dyadic inbreeding coefficient. Several examples of such pedigrees are provided by previous figures in this paper, for instance Figs. 1, 4, 8, 11 and 12.

Our work has important applications in the analysis of human pedigrees. Suppose we wish to check if a model depending on pedigree coefficients is sensitive to background inbreeding. With ribd this is straightforward, simply by assigning a suitable sequence of inbreeding values to the founders, and re-calculating the coefficients in each case. With earlier methods, the only alternative would be to model background inbreeding explicitly, by adding ever-larger genealogies to each founder. While theoretically possible to a certain extent, such an approach would be highly inefficient and ill-suited for studying the background inbreeding as a continuous variable.

The results of Sect. 4 show that ignoring background inbreeding may lead to serious bias in the computation of relatedness coefficients. Moreover, the effect is most pronounced in close relationships with small pedigrees. A striking conclusion one may draw from Fig. 4 is that a description of two individuals as “full siblings” or “half siblings”, may be quite misleading in terms of the closeness of their genetic relationship - even under the restriction that both individuals are noninbred. For example, two half siblings whose shared parent is completely inbred, are indistinguishable from an outbred parent-child pair.

While we have focused on autosomal relatedness coefficients in this paper, the ideas presented transfer easily to X-chromosomal coefficients. To our knowledge ribd is the only package with a complete set of functions for computing kinship and identity coefficients both for the autosomes and the X chromosome, as well as a variety of other single-locus and two-locus coefficients. Table 1 shows a comparison with the partially overlapping R packages kinship (Sinnwell et al. 2014), identity (Abney 2009) and XIBD (Henden et al. 2016), and the command-line tool PedKin (Kirkpatrick et al. 2018).

**Table 1:** Comparison of R packages computing relatedness coefficients. Abbreviations: A = autosomal; c = computable from other coefficients, but not available as a separate function; id = imported from the identity package; int = internal source code only, i.e., not available for end users; X = X-chromosomal

	Kinship		IBD (noninbred)		Identity		Generalised kinship	Selfing	Inbred founders
	A	X	A	X	A	X			
ribd	$\checkmark$	$\checkmark$	$\checkmark$	$\checkmark$	$\checkmark$	$\checkmark$	$\checkmark$	$\checkmark$	$\checkmark$
kinship2	$\checkmark$	$\checkmark$	–	–	–	–	–	–	–
identity/IdCoefs	c	–	c	–	$\checkmark$	–	–	$\checkmark$	–
XIBD	c	c	id	$\checkmark$	id	int	–	–	–
PedKin	$\checkmark$	–	–	–	–	–	–	–	$\checkmark$

We end the discussion by examining two possible extensions of the ideas presented in this work.

### Related founders

It is natural and interesting to seek a further extension of our approach, allowing pedigree founders to be not only inbred, but also related to each other. This would be particularly relevant for pedigrees in isolated populations, where the assumption of unrelatedness between all founders is unrealistic. However, the complexity of multi-person relatedness poses serious challenges for such an extension in full generality. For example, consider the algorithm in Sect. 3.1 for computing identity coefficients. If the founders are allowed to be related, then the boundary conditions (4) cease to hold, and must be replaced with formulas for $\varphi _{ab}$, $\varphi _{abc}$, $\varphi _{abcd}$ and $\varphi _{ab,cd}$, expressed by some coefficients describing the founder relationships. One might hope that these formulas only involved coefficients between each *pair* of related founders. Unfortunately this does not suffice in general, as shown by the following counter-example.

#### Example 12

**Fig. 13:**
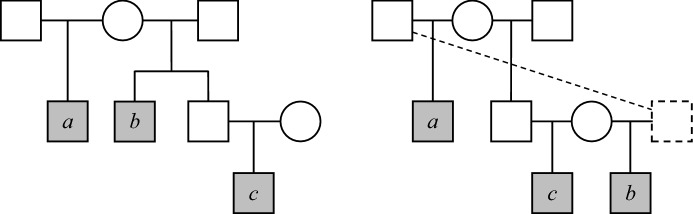
Two three-way relationships that have the same pairwise identity coefficients, but different generalised kinship coefficients. The dotted features in the pedigree to the right indicate a duplicated individual

Fig. 13 shows two pedigrees connecting three individuals *a*, *b* and *c*. We claim that these three-way relationships are identical in terms of the *pairwise* relationships, but have different generalised kinship coefficients. Indeed, in both pedigrees *a* is a half sibling of *b* and a half-uncle of *c*. The relationships between *b* and *c* are also inseparable, being uncle-nephew in the pedigree on the left hand side, and half siblings in the pedigree to the right (both of these have $\kappa = (\frac{1}{2}, \frac{1}{2}, 0)$). This proves the first part of the claim. For the last part, it is enough to observe that $\varphi _{abc} > 0$ in the left case (since all three may carry an allele originating from the (grand)mother), while $\varphi _{abc} = 0$ in the other (since there is no ancestor common to all of *a*, *b* and *c*).

To reiterate the point of Example 12, imagine a pedigree where *a*, *b* and *c* are founders, and we want to compute identity coefficients for some pair among their common descendants. Seeking a simple modification of the boundary conditions (4) to allow founder relatedness, we could try to express $\varphi _{abc}$ and the other generalised coefficients in terms of the pairwise relationships between *a*, *b* and *c*. Example 12 shows that any such attempt would be futile, suggesting that more complicated modifications would be necessary.

It may be the case that the modified boundary formulas require a complete characterisation of each quadruple of founders, i.e., the full set of 712 condensed identify coefficients for four individuals, as enumerated by Thompson (1974).

Nevertheless, the concept of founder relatedness may be worth investigating in simpler situations. One possibility is to restrict founder relatedness to *pairs* of founders, i.e., where different pairs are assumed to be unrelated. This approach was used by Lacy (2012) in the case of kinship coefficients, and may well be generalised to identity coefficients. This would allow extremely simple representations of many important relationships, including all noninbred relationships and many standard breeding schemes like brother-sister mating.

### Multi-locus coefficients

The study of relatedness coefficients extends naturally to multiple linked loci, by considering IBD distributions at two or more loci simultaneously. It is beyond our scope to review this rich subject here, instead we will simply point to the influential papers by Thompson (1988) and Weeks and Lange (1992) as good starting points.

**Fig. 14:**
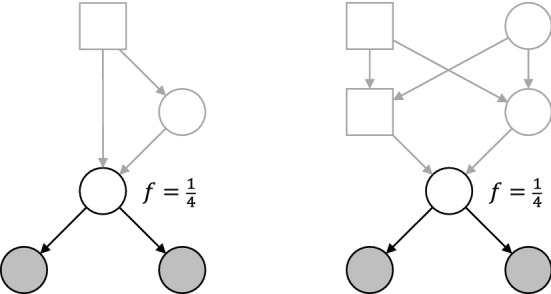
Half siblings with different constructions of the same inbreeding coefficient in the shared mother

**Fig. 15:**
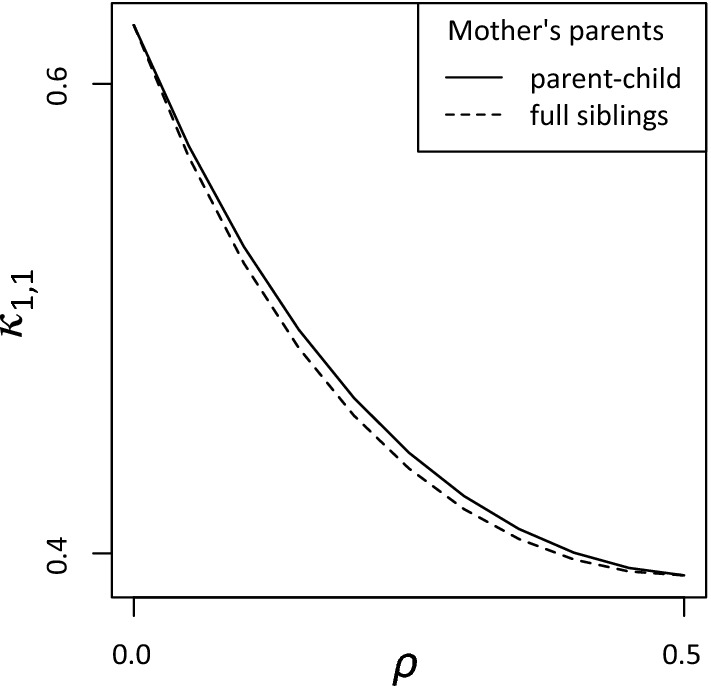
Two-locus IBD as a function of the recombination rate $\rho$, for the relationships in Fig. 14

Our use of inbred founders does not immediately apply to linked loci. The reason for this boils down to insufficient information carried by the (single-locus) inbreeding coefficient of an individual. To illustrate, consider the two cases of half sisters in Fig. 14, both with an inbreeding coefficient of $f = \frac{1}{4}$ in the shared mother. These relationships have the same single-locus IBD coefficients, $\kappa = (\frac{3}{8}, \frac{5}{8}, 0)$, but not the same *two-locus IBD* coefficient $\kappa _{1,1}(\rho )$. This is defined as the probability of sharing 1 allele IBD at each of two linked loci with recombination rate $\rho$. The graphs of $\kappa _{1,1}(\rho )$ corresponding to the two cases are given in Fig. 15.^1^ The fact that these graphs are not identical implies that, for the purpose of two-locus relatedness analysis, the genealogy of the mother cannot be compressed into the single coefficient $f = \frac{1}{4}$.

There is one important special case, however, where the (single-locus) inbreeding coefficient in fact captures the complete genetic constituency of the individual, namely when $f=1$. The choice of mating process used to produce a completely inbred individual, has no bearing on the distribution of IBD alleles among his or her descendants, even at linked loci. In particular, any recursive algorithm for computing multi-locus relatedness coefficients can in principle be modified to allow completely inbred founders.

## Conclusion

In this paper we have studied an extension of the conventional approach to pedigree analysis, in which we allow the assignment of inbreeding coefficients to the founders. The motivation is to enable a more compact representation of many pedigrees, while retaining sufficient information for exact computation of relatedness coefficients. This is particularly useful in cases where the true ancestries of certain pedigree members are unknown or unsuitable for computer modelling, such as completely inbred individuals. We believe that our implementation in ribd is the first software capable of computing identity coefficients in such pedigrees, even as simple as that in Fig. 1.

We also showed that pedigrees with inbred founders are especially potent in constructibility problems for relatedness coefficients. Previous solutions by Karigl (1984) required combinations of several infinite pedigrees in order to produce a given set of IBD coefficients. In contrast, our Theorem 8 guarantees that a finite pedigree suffices if inbred founders are allowed. The finiteness property is the crucial novelty here, since this opens up for computer analysis and practical applications.

The R package ribd is available from the CRAN repository (https://CRAN.R-project.org/package=ribd) and runs on all platforms. Importantly, ribd is part of the ped suite of packages for pedigree analysis, giving the user access to a large range of tools for creating, manipulating and visualising pedigrees, as well as likelihood computations and simulations. Pedigrees can be be loaded from text files in standard pedigree format, or made from scratch using built-in utility functions. Founder inbreeding is a core feature of the ped suite, allowing the ideas introduced in this paper to be explored in a variety of contexts.

## Footnotes

1 The data underlying these graphs were computed with the ribd package; code available upon request from the author.
